# Evidence of high bed net usage from a list randomization experiments in rural Gambia

**DOI:** 10.1186/s12936-020-03322-5

**Published:** 2020-07-13

**Authors:** Joe Brew, Margaret Pinder, Umberto D’Alessandro, Steven W. Lindsay, Caroline Jones, Elisa Sicuri

**Affiliations:** 1 ISGlobal, Hospital Clínic, Universitat de Barcelona, Barcelona, Spain; 2 VU University Amsterdam, De Boelelaan 1105, 1081 HV Amsterdam, The Netherlands; 3 Health Economics Group, Department of Infectious Disease Epidemiology, School of Public Health, Imperial College London, London, UK; 4 Medical Research Council Unit, The Gambia at the London, School of Hygiene and Tropical Medicine, PO Box 273, Banjul, The Gambia; 5 Department of Biosciences, Durham University, Stockton Road, Durham, DH1 3LE UK

**Keywords:** Malaria, List randomization, Long-lasting insecticidal nets, Gambia

## Abstract

**Background:**

Recording behaviours that have the potential to impact health can be doubly challenging if the behaviour takes place in private spaces that cannot be observed directly, and where respondents answer what they think the recorder may want to hear. Sleeping under a long-lasting insecticidal net (LLIN) is an important intervention for malaria prevention, yet it is difficult to gauge the extent to which coverage (how many nets are in the community) differs from usage (how many people actually sleep under a net). List randomization, a novel method which partially obscures respondents’ answers to sensitive questions, was employed to estimate LLIN usage in The Gambia.

**Methods:**

802 heads-of-household from 15 villages were recruited into a randomized controlled trial assessing the effect of a housing intervention on malaria. These houses were randomly assigned to a housing intervention versus control, with stratification by village so as to ensure balance between arms. From these, 125 households (63 intervention, 52 control) were randomly selected for participation in the list randomization experiment, along with 68 households from the same villages but which were not part of the housing improvement study, resulting in a total of 196 households for the list randomization experiment. Approximately half (n = 97) of the 196 study participants were randomly assigned to the control group and received a four-question list about non-sensitive behaviours; the intervention group (n = 99) received the same list, with the addition of one question on a sensitive behaviour: whether or not they had used a bed net the previous night. Participants were read the list of questions and then said how many of the statements were true. Bed net usage was estimated by calculating the difference in means between the number of affirmative responses between the two groups.

**Results:**

The mean number of affirmative responses in the control group was 2.60 of four statements (95% confidence interval, 95% CI 2.50–2.70), compared with 3.68 (95% CI 3.59–3.78) in the intervention group. Such difference (1.08; 95% CI 94.9–100%) suggests near universal bed net usage.

**Conclusions:**

Bed net usage by household heads in these rural villages was found to be high. Though not entirely unexpected given other studies’ estimates of high bed net usage in the area, the list randomization method should be further validated in an area with lower coverage.

## Background

From 2000 to 2015, the burden of malaria was reduced substantially in sub-Saharan Africa (SSA), with the prevalence of falciparum malaria declining by half and an estimated 663 million cases averted [1, 2]. This extraordinary achievement is due to the massive deployment of long-lasting insecticidal nets (LLINs), indoor residual spraying and prompt, effective treatment. Despite these gains, progress has stalled recently, with malaria cases rising slightly from 210 million in 2010 to 219 million in 2017 [2].

Scale up of LLINs and prompt and effective case management [3] were factors in averting 68% of cases [1]. LLINs protect users by providing a physical barrier to night-time biting mosquitoes and by killing mosquitoes upon contact. At around $7 per net distributed, they are a highly cost-effective intervention [3]. LLIN coverage in SSA has never been higher, with 80% of households having at least one net in 2016, and 43% of households having one or more nets for every two people [2]. However, there are concerns about how ‘coverage’ is measured and the extent to which high LLIN coverage translates to high usage [4].

Coverage is defined as the proportion of households with at least one LLIN for every two occupants [5], a metric that can be verified by counting the number of nets compared to the number of sleeping places. However, assessing bed net use, i.e., whether an individual actually sleeps under a net, is more difficult than assessing coverage. For example, while a household’s number of LLINs might surpass the threshold defined for coverage, household residents’ actual use may diminish after mass distribution campaigns due to product “wear and tear” [6], changes in use by season, social events, raised ambient temperature, and other factors. Assessments of LLIN usage often occur immediately after they are distributed, which may overestimate usage rates that are likely to decline over time. For example, a recent multi-country study suggested a 50% reduction in usage in the 23 months following LLIN distribution [7]. Perhaps of greater concern is the difficulty of estimating LLIN usage without directly observing people sleeping; studies usually rely on questionnaires and/or observing net(s) in a house. A recent meta-analysis estimated that self-reported rates of LLIN use were 13.6% greater than directly observed rates [8]. The extent of the gap between observed and reported usage is highly variable by country and social group [9].

The existence of this gap suggests that LLIN usage is potentially a sensitive behaviour. Reporting sleeping under a net is likely to be biased, since most recipients have been told that the net is protective. As a matter of politeness, or perhaps even fear of negative repercussions, respondents may say they have used the net, even when they have not. One method to reduce social desirability bias is list randomization [10, 11]. In a list randomization experiment, participants are divided into “control” and “treatment” groups. The control group is given a series of yes/no questions about everyday activities (communication, transportation, eating, and work), but instead of answering each question individually, participants simply tally the number of “yes” responses and report that number to the researcher. The experimental group is provided with the same questions, but with an additional question on a sensitive behaviour. Using list randomization, participants from both groups are able to obscure their item-specific responses from the researcher, but the data generated from the process allows for aggregate comparison between the groups, with the difference in total “yes” items approximating the population-level “yes” prevalence of the item in question.

List randomization has previously been used to reduce data bias pertaining to sensitive topics, such as personal finance [12], intimate partner violence [13], illegal migration [14], and attitudes regarding homosexuality and gender [15]. Many studies find higher rates of socially-sensitive behaviours via list randomization than direct questioning, suggesting that list randomization could be an effective tool for eliciting unbiased responses pertaining to socially-sensitive behaviours [16]. However, when Haber and colleagues compared list randomization responses to assess HIV status and sexual behaviour with known individual-level statuses, the former performed poorly [17]. In other words, though list randomization may be a useful method for gauging behaviours subject to social desirability bias, other biases may come into play. Despite its relevance to public health campaigns and its sensitive nature, there are no published reports on list randomization applied to the question of LLIN use. Thus, the present study employed list randomization to estimate LLIN use in an area of seasonal malaria transmission.

## Methods

### Study location

The study was carried out in the Upper River Region (URR) of The Gambia (13.12 N, 14.1 W). The URR is an area of open Sudanese savannah divided into north and south banks by the River Gambia. The climate consists of a long dry season from January to June, with rainfall occurring between June–July and October. Most clinical malaria cases are observed in October–December [18]. The Gambia has a long tradition of bed net use, particularly in rural areas [19].

### Study design

The list randomization experiment was part of a larger study on housing improvement and malaria. 91 villages were enrolled in a two-armed, household-clustered, randomized controlled trial using a block design (with the village as the block and households as the randomization unit) to assess the impact of housing improvement on malaria outcomes. Further details on the randomization and clustering design are available in the study protocol [20].

Fifteen of the 91 trial villages were randomly selected for an ancillary longitudinal socio-economic study, stratified by riverbank (north vs south), ethnic group (with the purposely selection of one, Jagajari, for being the only Serrehule village in the trial), and size. The list randomization experiment was included in one of the four rounds composing the socio-economic study. 196 households were selected for participation in the list randomization experiment. The selection included both thatched-roof and metal-roof houses. The list randomization component took place from the end of November 2017 (approximately peak transmission) to mid-January 2018 (when mosquito density was low). A total of 1513 LLIN were distributed by the Roo*Pf*s trial in 2015/2016 before the trial started and a national campaign carried out by the National Malaria Control Programme (NMCP) also distributed free LLINs in July 2017, a few months before the list randomization experiment.

Enrolled household heads were randomly assigned using a simple randomization script in Visual Basic to one of two “question lists”: 97 received the control questionnaire and 99 the experiment questionnaire. The control questionnaire contained four questions about daily activities; the experiment questionnaire contained the same questions, with an additional question asking whether the participant slept under a LLIN the previous night (Table 1). The order of statements in all questionnaires was randomized at the individual level, thus, every household head had his or her own randomly assigned questionnaire. The maximum number of statement combinations [N * (N − 1)] was 12 (4 * 3) in the control group and 20 (5 * 4) in the experimental group. Therefore, 12 and 20 different typologies of questionnaires were randomized to the households.

**Table 1:** List randomization question list

Control	Experiment
I used a telephone yesterday	I used a telephone yesterday
I used transportation other than walking yesterday	I used transportation other than walking yesterday
I ate benachin yesterday	I ate benachin yesterday
I worked yesterday	I worked yesterday
	I slept under a mosquito net last night

The statement in bold represents the “experimental” item. “Benachin”, also known as “jollof rice”, is a staple meal in the Gambia

A trained field assistant obtained written, informed consent from the identified household heads, and administered the question list to them using their preferred local language in their household compound. Participants were asked not to address individual statements, but instead to count on their fingers (held behind their backs, so as to block the view of the interviewer) the number of statements which were true for them. The reported number of true statements (0 to 4 for the control group, 0 to 5 for the experimental group) was recorded.

The sample size of 196 participants, chosen based on operational limits (budget and field assistant availability), was sufficient for the calculation of a 95% confidence interval with a margin of error of 5% on a LLIN usage point estimate of 85%.

### Ethics

Participants were enrolled into the study provided they gave their full and informed written consent. The study was approved by the Gambia Government and Medical Research Council’s joint ethics committee.

## Results

196 household heads freely agreed and completed written informed consent to participate in the list randomization experiment. Comparison of important variables were similar in both experimental groups (Table 2).

**Table 2:** Characteristics of study groups

Variable	Category	List randomization group	
		Control	Treatment
Housing improvement intervention	Intervention	30 (30.9%)	33 (33.3%)
	Control	34 (35.1%)	28 (28.3%)
	Not in study	33 (34.0%)	35 (35.4%)
Ethnicity	Fula	60 (61.9%)	61 (61.6%)
	Mandinka	33 (34.0%)	33 (33.3%)
	Sarahule	4 (4.1%)	4 (4.0%)
	Unknown	0	1 (1.0%)
Village size	Large	33 (34.0%)	33 (33.3%)
	Small	64 (66.0%)	66 (66.7%)
River bank	North	31 (32.0%)	33 (33.3%)
	South	66 (68.0%)	66 (66.7%)
Gender	Male	52	50
	Female	45	45
	No response/missing	0	4
Age	Mean	44.7	44.6
	Standard deviation	8.12	8.25

### Unbiased bed net coverage estimates via difference in means

The mean number of agreements in the control group was 2.60 (95% confidence intervals CI 2.50–2.70) of four questions. In the experiment group, the mean number of agreements was 3.68 (95% CI 3.59 to 3.78) of five questions. The difference of 1.1 is indicative of 100% bed net usage among the study population. A 95% confidence interval of the difference was estimated via t-test to be 94.9–100% (t = 15.67, p < 0.001). Notably, no respondents reported the minimum (zero) or maximum (four or five, depending on the group) number of activities from the list.

## Discussion

Though the individual bed net status of participants is unknown to the field assistant and researcher, aggregating by group allows one to estimate the percentage of participants who agreed with the experimental statement, since in all other aspects the participants’ responses should converge towards being identical due to random assignment. As with other list randomization experiments, it was assumed that (i) the introduction of the experimental item would not affect responses to other items and (ii) that the degree of accuracy to non-experimental items would be similar across groups (i.e., the “no design effect assumption” [21]). Accordingly, the difference between the average number of agreements in the two groups, with uncertainty quantified by a t-test, should reflect the proportion of participants in the experiment group which agreed with the experimental statement.

The experiment indicates very high bed net use by household heads in rural eastern Gambia (94.9–100%). The estimate of virtually universal LLIN usage is significantly higher than previous published figures on LLIN coverage obtained via other methods [18]. For example, net coverage in 2010 in the Upper River Region, including the urban areas, was only 68% [22], and a 2017 national survey showed that 75.7% of the Upper River Region population had de facto access to a LLIN [23].

The context may partially explain this finding: the study took place at the end of the malaria transmission season, a LLIN distribution campaign had recently been carried out, and recent information and sensitization campaigns were taking place as part of the Roo*Pf*s trial [20]. Also the trial setting may have had associated behavioural effects. The high usage estimate was also found in the main trial study, in which 93.9% of children living in houses enrolled in the trial were reported to have slept under an LLIN (Pinder et al. pers. commun.) Even though this latter figure referred to children and was obtained via a direct questionnaire to their caretaker, it suggests high usage in this setting.

Though the finding of high bed net usage is plausible given the context, this study has four important limitations in terms of generalizability: (1) the study lacked any evidence-based method for validating responses (i.e., direct observation); (2) the study took place in an area where a great deal of health research had already taken place, opening the door to the possibility that our population was not representative of West Africa or even The Gambia especially since they had already been sensitized to malaria-related issues given their participation in the housing improvement trial; (3) though the sample size was sufficient for an overall assessment of bed net coverage based on a two-group comparison, there was not sufficient statistical power to identify the potential determinants of bed net use, such as ethnicity, age, or socioeconomic status; and (4) there were no responses with the minimum (0 “yes” items) or maximum (4 or 5 “yes” items, depending on the arm) affirmations, suggesting that there may have been some unanticipated biases despite the method.

This final point merits further exploration. A potential cause of edge-avoidance in responses may be the consequence of poor item selection (i.e., items which did not provoke heterogeneity in responses). This is unlikely given that all four non-experimental items were chosen with the intention of neither being universal nor universally avoided. A more likely cause is that the list randomization method itself may not be an effective elicitation tool in certain contexts. Haber and colleagues, who also used the technique of having respondents finger-count affirmative items behind their back, found that list randomization did not correlate strongly with known ground-truth, and actually performed worse than direct questioning in some areas [17]. This is consistent with the findings by Arentoft and colleagues that list randomization did not result in higher frequencies of socially-sensitive behaviour reports compared to direct questioning [24]. Arentoft suggests that the reason for unexpectedly low affirmative responses to list randomization questionnaires might be that some participants may detect the “quasi-covert nature” of the study; Haber’s main hypothesis explaining poor list randomization is “cognitive difficulty”—that is, the unusual and non-physical nature of tallying up responses may be confusing. Both of these factors may have contributed to this study’s high usage finding. In regards to edge-avoidance, it may simply be the case that participants did not want to reveal item-specific responses to *any* of the behaviours. Since an all “yes” or all “no” questionnaire means each item-specific response is known to the interviewer, participants may have gravitated towards non-edge responses so as to obscure all items. This is consistent with the fact that edge-avoidance took place in the experimental arm and the non-experimental arm (i.e., those administered the questionnaire without the bed net item).

Unlike HIV status [17, 24], which can be validated via laboratory test, the nature of sleeping under a LLIN makes ground-truth validation unfeasible. Even if study participants consented to being observed directly while sleeping (via camera or direct observation), this would undoubtedly introduce an even greater degree of social desirability bias, not to mention important concerns about privacy. A movement logger could theoretically be used as a less invasive and more accurate validation tool, but the awareness of the logger itself might also bias results, since one can assume that an individual is more likely to use an LLIN if they know their use is being monitored. Though list randomization is administratively complicated and subject to biases, it is a method which merits further research since alternatives—direct questioning, direct observation, mechanical monitoring—are subject to perhaps greater biases. For the question of LLIN usage, future research using the list randomization method should be directed in two areas: (1) to validate the method itself by (a) attempting an objective validation, (b) gauging seasonal variability in responses and (c) reproducing in other contexts, particularly where bed nets use has lower tradition than in The Gambia and far in time from recent bed net distributions; and (2) to better understand the possible social or cognitive biases that might explain edge-avoidance in responses.

## Conclusion

List randomization offers a novel approach for exploring LLIN use in study communities, since the use of a LLIN can be considered a socially desirable behaviour and, therefore, subject to social desirability bias. The results of this list randomization experiment suggest very high LLIN usage among household heads in a rural area of a region of The Gambia with high coverage. High usage in a context of high coverage would be good news for public health practitioners worried about disuse and misuse, and is consistent with previous research showing high LLIN use following distribution campaigns [25–27]. Though list randomization has been shown to be a useful tool for eliciting sensitive behaviours in other contexts, for the specific case of LLIN usage further research is needed.

## Data Availability

The datasets generated and analysed during this study are not publicly available since they include identifiable protected health information of a sensitive nature. Researchers interested in accessing the data can contact the authors, to put them in contact with the ethics committee of the Medical Research Council Unit The Gambia at the London School of Hygiene and Tropical Medicine, PO Box 273, Banjul, The Gambia and will facilitate access to the data in the case of ethical approval being obtained.
